# Metabolic reprogramming, oxidative stress, and mitophagy in JSRV Env-transformed BEAS-2B cells: insights from integrated transcriptomics and metabolomics

**DOI:** 10.1186/s12864-026-13125-8

**Published:** 2026-06-29

**Authors:** Xujie Duan, Wenjing Lan, Rui Liu, Pei Zhang, Sixu Chen, Yufei Zhang, Liang Zhang, Huiping Li, Shuying Liu

**Affiliations:** 1 College of Veterinary Medicine, Inner Mongolia Agricultural University, Hohhot, 010018 China; 2 Inner Mongolia Key Laboratory of Veterinary Fundamentals and Disease Prevention and Control for Herbivorous Livestock, Inner Mongolia Agricultural University, Hohhot, 010018 China; 3 Inner Mongolia Key Laboratory of Basic Veterinary Medicine, Inner Mongolia Agricultural University, Hohhot, 010018 China

**Keywords:** Jaagsiekte sheep retrovirus, Transcriptomics, Metabolomics, Mitophagy, Metabolic reprogramming

## Abstract

**Background:**

Jaagsiekte sheep retrovirus (JSRV) causes ovine pulmonary adenocarcinoma (OPA), and its pathogenesis is primarily mediated by the viral envelope (Env) protein. However, the detailed oncogenic mechanisms underlying JSRV infection remain incompletely understood. In this study, we integrated transcriptomic and metabolomic analyses to characterize JSRV Env-induced alterations in human bronchial epithelial BEAS-2B (cells).

**Results:**

BEAS-2B cells transfected with the pcDNA4.0myc-his-JSRV-*env* plasmid, those transfected with the empty pcDNA4.0myc-his vector, and untreated BEAS-2B cells served as the experimental, negative control, and blank control groups, respectively. Transcriptomic analysis identified a total of 2733 differentially expressed genes (DEGs). Specifically, relative to the blank and negative control groups, 1178 genes were upregulated and 307 were downregulated in the JSRV-env group. These DEGs were significantly enriched in pathways related to altered cellular energy metabolism, cell cycle regulation, and oncogenic signaling. Metabolomic analysis revealed 451 differentially expressed metabolites (DEMs), with 192 detected in positive ion mode and 259 in negative ion mode. Compared with both control groups, the JSRV-env group exhibited 33 upregulated and 46 downregulated DEMs in positive ion mode, as well as 22 upregulated and 55 downregulated DEMs in negative ion mode. These DEMs were significantly enriched in pathways such as cellular metabolism, purine metabolism, amino acid metabolism, and the tricarboxylic acid cycle. Notably, cellular and mitochondrial energy metabolism pathways were closely linked. Analyses of mitochondrial- and mitophagy-related genes, alongside an integrated transcriptomic and metabolomic evaluation of their interactions, suggested mitochondrial damage and the potential activation of mitophagy. Furthermore, JSRV Env-transformed BEAS-2B cells exhibited elevated reactive oxygen species, decreased mitochondrial membrane potential, and abnormal mitochondrial morphology—characterized by swelling, as well as fragmented, dissolved, or disappearing cristae—along with the presence of myelin-like mitochondrial lesions and mitophagosomes. Mitochondrial and lysosomal probe co-localization further confirmed mitochondrial degradation in the transformed cells.

**Conclusions:**

Overall, these results highlight the potential involvement of altered mitochondrial energy metabolism and mitophagy in JSRV Env-induced BEAS-2B cell transformation. These findings offer novel insights into the mechanisms of viral oncoproteins, cellular metabolic reprogramming, and mitophagy, while identifying potential targets for understanding JSRV pathogenesis.

**Supplementary Information:**

The online version contains supplementary material available at https://doi.org/10.1186/s12864-026-13125-8.

## Background

Jaagsiekte sheep retrovirus (JSRV), a pathogen belonging to the *Retroviridae family*, *Orthoretrovirinae subfamily*, and *Betaretrovirus genus*, causes ovine pulmonary adenocarcinoma (OPA) which seriously endangers the efficient and healthy development of the sheep-raising industry [1]. As a lung-tropic retrovirus, JSRV induces malignant proliferation of lung epithelial cells, including type II alveolar epithelial cells and non-ciliated bronchiolar Clara cells, forming invasive tumor lesions in sheep. JSRV oncogenicity mainly depends on the envelope protein (Env), encoded by the *env* gene, which acts as the primary viral oncogene. Env comprises two subunits: the surface protein and the transmembrane protein [2]. Upon binding to specific host receptors, these subunits initiate oncogenic signaling cascades within host cells, leading to malignant transformation [3]. The current bottleneck in JSRV research is the lack of an effective in vitro culture system for the wild-type virus, which greatly limits mechanistic studies. Therefore, JSRV-*env* overexpressing cell models are widely used to study the in vitro mechanisms of JSRV.

Natural JSRV infection features a prolonged incubation period of up to 3–4 years before clinical onset in sheep, a chronicity largely driven by its long terminal repeat (LTR), which contains promoter and enhancer elements that interact with host transcription factors to gradually initiate viral gene expression. Owing to this long incubation period and susceptibility to interference from other pathogens in vivo, in vitro cell models have become indispensable tools for overcoming this research bottleneck [4]. Consequently, these models typically employ the cytomegalovirus (CMV) promoter to drive JSRV env expression, bypassing the slow LTR-mediated process and enabling the rapid observation of Env-induced malignant transformation [5]. The transforming properties of JSRV Env have been confirmed in various cell lines, including NIH3T3, 208 F, DF-1, MDCK, RK3E, and BEAS-2B cells. However, the underlying mechanisms are highly cell type-specific [6]. Unlike the cytoplasmic tail-dependent activation of the PI3K-Akt pathway observed in rodent fibroblasts, Env-mediated transformation in BEAS-2B cells is strictly dependent on the receptor Hyaluronidase 2 (Hyal2) [7]. Under physiological conditions, Hyal2 constitutively binds to and inhibits the receptor tyrosine kinase RON. JSRV Env binding triggers Hyal2 degradation, leading to RON release and the subsequent activation of downstream oncogenic pathways, such as PI3K-Akt and MAPK [8]. Given the high expression of RON in human lung adenocarcinoma (LUAD), this Hyal2-RON axis provides a highly relevant paradigm for understanding human lung carcinogenesis. Furthermore, beyond viral oncogenes, BEAS-2B cells are widely recognized as a robust in vitro model for studying malignant transformation induced by diverse environmental and biological carcinogens, such as tumor-derived exosomes and cigarette smoke extract [9, 10]. Consequently, BEAS-2B cells serve as an exceptional reference model for studying both JSRV Env and LUAD [11]. Although the initial signaling events have been mapped, the comprehensive molecular and metabolic reprogramming underlying JSRV Env-induced malignant transformation remains largely unknown.

To study the pathogenic mechanism of JSRV, we previously explored the ultrastructural lesions of naturally infected JSRV lung tissues and found closely arranged proliferating epithelial cells, mostly comprising type II alveolar epithelial cells with lamellar bodies and microvilli. The mitochondria in type II alveolar epithelial cells were significantly swollen, with dissolution and fragmentation of the cristae, indicating severe mitochondrial damage. A large number of autophagosomes and mitophagosomes were observed, suggesting the activation of autophagy as a cellular protective response to clear damaged organelles [12]. We then performed transcriptome sequencing (RNA-Seq) on lung tissues naturally infected with JSRV, revealing that differentially expressed genes (DEGs) were mainly involved in the activation of signaling pathways such as PI3K-Akt, Hippo, and MAPK [13]. Given the possible involvement of multiple pathogen infections and varying disease courses in natural OPA infection, significant genomic differences may be present in lung tissues. Therefore, a well-controlled in vitro cell model may help uncover the key genes specifically involved in the pathogenic mechanism of JSRV Env.

RNA-Seq, a core technology in systems biology, can comprehensively capture the transcriptome information of cells under specific conditions and reveal dynamic changes in gene expression regulatory networks [14]. Currently, the transcriptomics of JSRV Env-transformed BEAS-2B cells remain unexplored; therefore, the genes, pathways, and regulatory networks that undergo significant remodeling during JSRV Env-induced cell transformation remain unknown. This greatly limits our in-depth understanding of the tumorigenic mechanism of JSRV and hinders research progress in the development of early diagnostic markers and therapeutic targets. As the final downstream level of the genome and transcriptome, dynamic changes in the metabolome directly reflect functional phenotypic alterations in cells under the influence of JSRV Env and serve as a crucial link between gene regulation and malignant cell phenotypes. Minor changes in gene expression may be amplified through enzymatic reaction cascades, ultimately manifesting as significant metabolite differences. These differences are often directly related to malignant biological behaviors such as cell proliferation and invasion [15]. Capturing the types and trends of differentially expressed metabolites (DEMs) in transformed cells allows for the identification of abnormal nodes in core metabolic pathways, including energy metabolism, amino acid metabolism, and lipid remodeling. Integrating these metabolomic findings with transcriptomic data enables the construction of regulatory networks involving DEGs and DEMs, thereby providing a more comprehensive understanding of the tumorigenic mechanism of JSRV Env.

Therefore, in the present study, we performed an integrated transcriptomic and metabolomic analysis of BEAS‑2B cells transfected with JSRV‑*env*, using appropriate blank and negative controls to identify robust signatures of gene expression and metabolite changes. By combining Gene Ontology and KEGG pathway enrichment analyses, we explored the biological functions and signaling pathways perturbed during Env‑induced transformation. Prompted by the mitochondrial ultrastructural lesions observed in naturally infected lung tissues, which strongly suggested the activation of mitophagy, we specifically focused on genes and metabolites related to mitochondrial damage, mitophagy, and energy metabolism to further delineate the mechanisms underlying JSRV Env‑driven malignant transformation. Overall, this study aimed to elucidate the molecular landscape of JSRV Env‑induced transformation from multiple perspectives—including transcriptional reprogramming, pathway activation, metabolic rewiring, and intracellular homeostasis regulation—thereby providing a theoretical basis for understanding the essence of malignant transformation and identifying potential early diagnostic or therapeutic targets.

## Methods

### Culture and plasmid transfection of BEAS − 2B cells

In the present study, human bronchial epithelial BEAS-2B cells, purchased from the Cell Bank of the Chinese Academy of Sciences (Shanghai, China), were cultured in Dulbecco’s Modified Eagle Medium (Gibco, Thermo Fisher Scientific, Waltham, MA, USA) supplemented with 10% fetal bovine serum (Shanghai ExCell Biology Inc., Shanghai, China), 100 IU/mL penicillin, and 100 µg/mL streptomycin at 37 °C in a 5% CO₂ incubator. All experiments were performed with cells between passages 4 and 8. The eukaryotic expression plasmids pcDNA4.0myc-his-JSRV-*env* was preserved in our laboratory. JetOPTIMUS transfection reagent (Polyplus-transfection SA, Illkirch, France) was used for plasmid transfection, following the manufacturer’s protocol. Cells without any treatment were designated as the blank group, BEAS-2B cells transfected with the pcDNA4.0myc-his plasmid served as the negative control group, and BEAS-2B cells transfected with the pcDNA4.0myc-his-JSRV-*env* plasmid were assigned to the experimental group (JSRV-*env*). Total RNA was extracted from cells at 24 h, 48 h, and 72 h post-transfection (hpt), respectively.

### Total RNA extraction and reverse transcription‑quantitative PCR

Total RNA was extracted from nine samples using TRIzol (TAKARA, Kusatsu, Shiga, Japan) according to the manufacturer’s protocol and dissolved in RNase-free water. The concentration and purity of RNA were verified by NanoDropND-1000 (Thermo Fisher Scientific, Wilmington, DE, USA) and agarose gel electrophoresis. RNA integrity was assessed using a Bio-analyzer 2100 system (Agilent Technologies). Total RNA was reverse transcribed into cDNA using a HiScript III RT Super Mix for qPCR with gDNA Eraser (Vazyme, Nanjing, Jiangsu, China). The synthesized cDNA was quantified with a ChamQ Universal SYBR qPCR Master Mix (Vazyme, Nanjing, Jiangsu, China) on an ABI 7500 sequence detection system (Applied Biosystems), following the manufacturer’s protocol. The Ct value of each target was obtained by calculating the arithmetic mean from technical triplicates. The relative expression levels of mRNA were calculated with the 2^−ΔΔCt^ method. The Primer sequences are shown in additional file 1: Table S1.

### Extraction of total proteins and Western blot analysis

Total protein from cells was extracted using the total protein extraction kit (Sangon Biotech, Shanghai, China) and quantified by the BCA method. The protein samples were mixed with loading buffer and denatured at 100 °C for 10 min. Equal amounts of protein (25 µg) were separated by 10% SDS-PAGE and transferred onto a PVDF membrane (Merck, Germany). After blocking with 5% skimmed milk for 2 h at room temperature, the membrane was incubated with the primary antibody against the His-tag (Proteintech, Wuhan, China)(1:5000) at 4 °C for 12 h, followed by incubation with an HRP-conjugated secondary antibody for 90 min at room temperature. The membrane was developed using an ECL chemiluminescence substrate and detected using a chemiluminescence imaging system (Tanon, Shanghai, China).

### Cell immunofluorescence

The JSRV Env antibody was preserved in our laboratory. After transfecting BEAS-2B cells with the pcDNA4.0myc-his-JSRV-*env* plasmid for 48 h, cells were rinsed with DPBS, fixed with paraformaldehyde (Solarbio, Beijing, China) and permeabilized with Triton X-100 (Solarbio, Beijing, China). Following blocking with BSA (Merck KGaA, Darmstadt, Germany), cells were sequentially incubated with JSRV Env primary antibody (1:300) at 4 °C overnight and CoraLite488-conjugated Goat Anti-Rabbit IgG(H + L) (1:150) (Proteintech, Wuhan, China) at 37 °C. After staining the cell nuclei with DAPI (Solarbio, Beijing, China) in the dark, images were acquired using a laser scanning confocal microscope (Carl Zeiss AG, Baden-Wurttemberg, Germany).

### RNA-seq analysis

Total RNA was extracted from BEAS-2B cells 48 h after transfection with the pcDNA4.0myc-his-JSRV-*env* plasmid. After total RNA passed quality control assessment, transcriptome libraries were constructed using the VAHTS Universal V6 RNA-Seq kit (Vazyme, Nanjing, Jiangsu, China). Sequencing was performed on the Illumina NovaSeq 6000 platform (150 bp paired-end mode), yielding approximately 50 million raw reads per sample. Clean reads were retained after quality filtering with Fastp (v0.18.0) and aligned to the human reference genome using HISAT2 (v2.1.0). StringTie (v1.3.4) was used to assemble the mapped reads of each sample and merge them into a new transcriptome, while RNA-Seq by Expectation Maximization was employed to quantify the expression levels of all genes in each sample. Clean reads were aligned to the human ribosomal database using the short-read aligner Bowtie2 (v2.28), ribosome-mapped reads were removed, and the remaining unmapped reads were retained as new clean reads for subsequent transcriptome analysis. Genes showing zero expression across all samples were excluded. DEGs between group of BEAS − 2B cells transfected with JSRV-*env*, blank and control were identified using DESeq2 (R v4.3.2), applying thresholds of |log₂ fold change (FC)| ≥1 and adjusted *p*-value < 0.05. Subsequently, Gene Ontology (GO) and KEGG pathway enrichment analyses were performed for the DEGs (Gene Ontology Consortium, http://geneontology.org; KEGG, http://www.kegg.jp/kegg).

### Extraction, detection and quantitative analysis of metabolites

Cell samples (1 × 10⁷) were extracted with a methanol: acetonitrile: water (2:2:1) solution, followed by protein precipitation, lyophilization, and reconstitution in acetonitrile: water (1:1). The extracts were separated on a Waters BEH Amide HILIC column using a gradient of mobile phase A (25 mM ammonium acetate/ammonia in water) and B (acetonitrile). Analysis was performed on a Thermo Orbitrap Exploris 480 mass spectrometer in both positive and negative ion modes, using a data-dependent acquisition method. This method consisted of a full MS scan (m/z 70-1200, 60,000 resolution) triggering HCD-MS/MS scans of the top 10 most intense ions (60,000 resolution, 15 s exclusion). To ensure data quality, samples were randomized with a quality control (QC) injection inserted every 10 samples.

### Integrative transcriptome and metabolome analysis

Key genes and metabolites potentially mediating the pathogenic mechanism of JSRV Env were identified via targeted screening. Associations were analyzed in the Omicshare Platform to identify potentially co-regulated genes and metabolites.

### Verification of RNA-seq data through quantitative reverse transcription polymerase chain reaction (RT-qPCR)

The RT-qPCR assay was performed as previously described. Primers used for validating RNA-Seq data are listed in Table S2, and primer information for downstream differential genes of metabolomic metabolites is provided in Table S3.

### Detection of mitochondrial damage and mitophagy markers following JSRV Env-induced transformation of BEAS-2B cells

Cellular reactive oxygen species (ROS) levels were quantified using the DCFH-DA probe. Blank/negative controls, JSRV-transformed BEAS-2B cells, and Rosup-treated positive controls (10 µM, 30 min, kit-provided positive control) were incubated with DCFH-DA (1 mL, 37 °C/5% CO₂, 20 min), washed, and imaged via confocal microscopy (488 nm excitation) [16]. Mitochondrial membrane potential (MMP) was evaluated using the JC-1 dye. Blank/negative controls, JSRV-transformed BEAS-2B cells, and Carbonyl cyanide m-chlorophenyl hydrazone (CCCP) -treated positive controls (10 µM, 20 min) were incubated with JC-1 working solution (1 mL, 37 °C, 20 min), washed with JC-1 buffer, and imaged. Red fluorescence (585 nm ex.) indicates high MMP, while green fluorescence (514 nm ex.) indicates mitochondrial depolarization [17]. All groups were imaged post-medium replacement. Ultrastructural changes were examined by transmission electron microscopy (TEM). Cells were fixed with 2.5% glutaraldehyde, post-fixed with 1% osmium tetroxide, dehydrated through an ethanol gradient, and embedded in resin [18]. Ultrathin sections were stained with uranyl acetate and lead citrate before imaging with TEM to compare JSRV Env-transformed cells against controls and CCCP-treated cells. To detect mitophagy, mito-lysosomal colocalization was assessed. Cells were first incubated with Mito-Tracker Green (20 nM) and Hoechst 33,342 (10 µg/mL) at 37 °C for 45 min [19]. After washing, cells were subsequently incubated with Lyso-Tracker Red (50 nM) at 37 °C for 60 min. Following a final medium replacement, cells were imaged using a laser confocal microscope.

### Statistical analysis

Data were analyzed with the GraphPad Prism 8.1 software and expressed as the mean ± standard error of the mean. An unpaired t-test was used to compare the data corresponding to the single factor following the normal distribution between the two groups. Data corresponding to multiple factors were analyzed by two-way analysis of variance. Statistical significance was indicated by *P* < 0.05.

## Results

### Screening of the optimal transfection time for the pcDNA4.0myc-his-JSRV-env plasmid in BEAS-2B cells

**Fig. 1:**
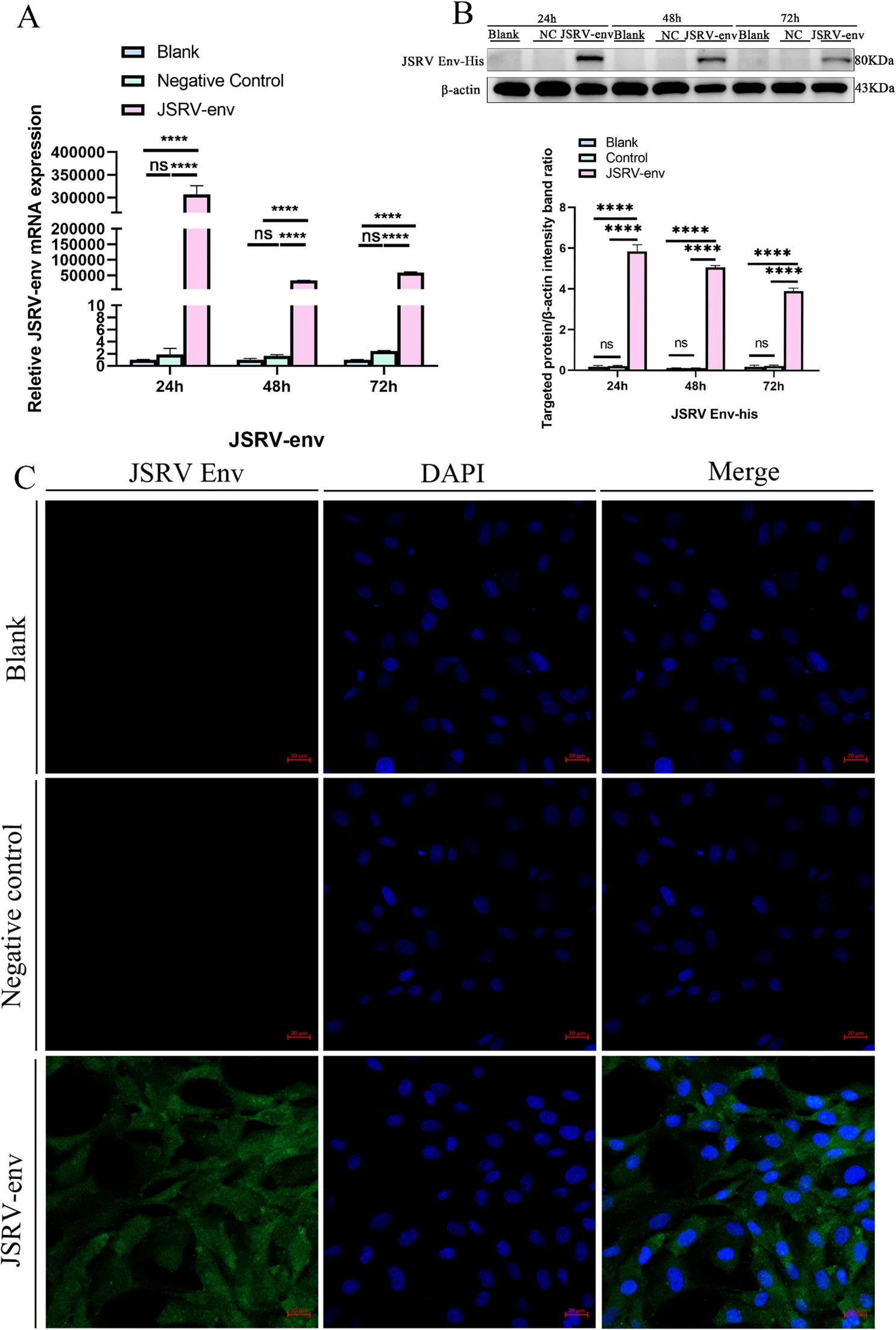
Determination of optimal transfection conditions for the pcDNA4.0myc-his-JSRV-env plasmid in BEAS-2B cells. **A** Relative mRNA expression levels of Jaagsiekte sheep retrovirus envelope (JSRV-env) in human bronchial epithelial cells (BEAS-2B) cells transfected with the pcDNA4.0myc-his-JSRV-env plasmid at 24, 48, and 72 hpt (**B**) Western blot analysis and quantification of the JSRV Env-His fusion protein (**C**) Confocal images indicating JSRV Env expression at 48 hpt Cells were immunostained with an anti-JSRV Env antibody (green). Nuclei were stained with DAPI (blue) (scale bar = 20 μm). P values were calculated using two-way analysis of variance. ^*^P < 0.05, ^**^P < 0.01, ^***^P < 0.001, ^****^P < 0.0001

To determine the optimal transfection duration for the pcDNA4.0myc-his-JSRV-*env* plasmid in BEAS-2B cells, we measured the mRNA levels of JSRV-*env* at 24, 48, and 72 h post-transfection (hpt). RT-qPCR analysis revealed that the mRNA expression of JSRV-*env* was significantly higher in the pcDNA4.0myc-his-JSRV-*env* transfected group than in the blank and negative control groups at all three time points. The relative expression levels peaked at 24 hpt and stabilized at 48 and 72 hpt (Fig. 1A). Consistent with the mRNA levels, the relative protein expression of JSRV Env exhibited a similar trend (Fig. 1B). Therefore, the stable expression time point of 48 hpt was selected for subsequent experiments. Immunofluorescence staining was performed to determine the JSRV Env expression levels in each group at 48 hpt. Green fluorescent protein was detected in BEAS-2B cells transfected with the pcDNA4.0myc-his-JSRV-*env* plasmid (Fig. 1B). These results indicated that transfection of BEAS-2B cells with the pcDNA4.0myc-his-JSRV-*env* plasmid for 48 h was suitable for subsequent studies.

### Overall evaluation of RNA-Seq data

**Fig. 2:**
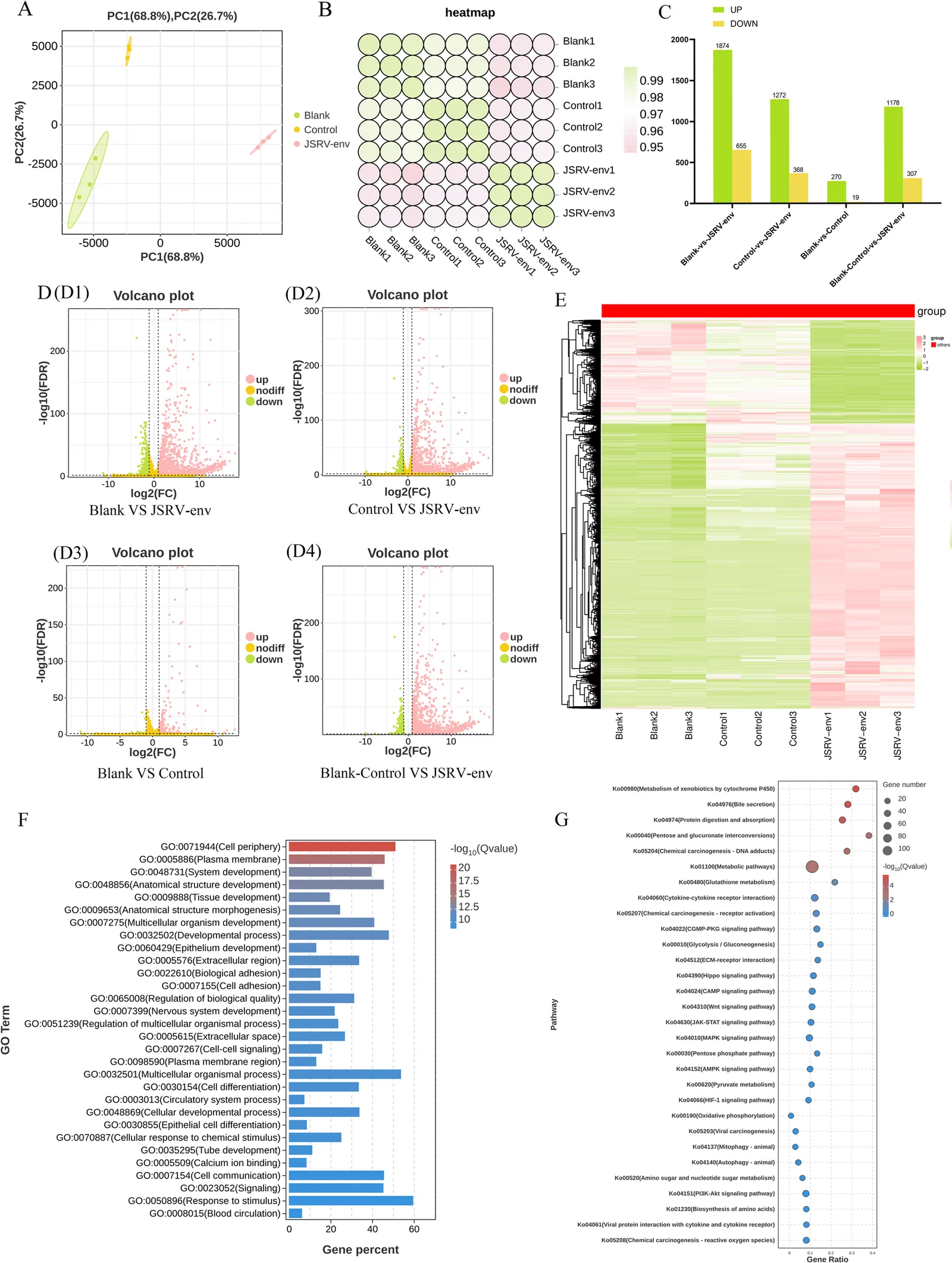
Transcriptomic analysis of JSRV Env-transformed BEAS-2B cells. **A** Principal component analysis results of samples (**B**) Sample correlation heatmap (**C**) Column chart of differentially expressed genes (DEGs) (**D**) Volcano plot of DEGs (D1. Blank vs. JSRV-env; D2. Control vs. JSRV-env; D3. Blank vs. Control; D4. Blank-Control vs. JSRV-env) (**E**) Cluster heatmap of DEGs (**F**) Gene Ontology (GO) enrichment analysis of DEGs (**G**) Kyoto Encyclopedia of Genes and Genomes (KEGG) enrichment analysis of DEGs. Abbreviations: JSRV, Jaagsiekte sheep retrovirus; BEAS-2B, human bronchial epithelial cells; Env, envelope

To investigate the potential mechanism of JSRV Env-induced transformation of BEAS-2B cells, we performed transcriptome analysis. High-quality sequencing was achieved with a data yield of more than 6 Gb per sample and an average Q20 score exceeding 97% for all samples. On average, 97.10% of the reads were successfully aligned with the reference genome (Table S4). Principal component analysis (PCA) revealed distinct clustering of the three groups, indicating significant transcriptomic differences (Fig. 2A). Correlation coefficients between the groups ranged from 0.949 to 0.991, indicating characteristic differences between groups, which supported their suitability for subsequent analytical experiments (Fig. 2B).

### DEGs analysis

In this study, we identified a total of 2,733 DEGs across the blank control, negative control, and JSRV-env-transfected BEAS-2B cell groups. The JSRV-env-transfected group exhibited the most pronounced transcriptional changes, with 1,874 upregulated and 655 downregulated genes compared to the blank control, and 1,272 upregulated and 368 downregulated genes compared to the negative control. Notably, relative to both control groups, the JSRV-env-transfected cells shared 1,178 commonly upregulated and 307 commonly downregulated genes. These findings indicated that JSRV-env transfection induced extensive alterations in the gene expression profile of BEAS-2B cells (Fig. 2C).

Volcano plots visualized the statistical significance and magnitude of these changes, revealing a substantial number of DEGs with a greater proportion of upregulated than downregulated genes (*P* < 0.05, |log₂Fold Change| > 1) (Fig. 2D). Furthermore, hierarchical clustering clearly segregated the three groups into distinct clusters based on their unique expression profiles (Fig. 2E), confirming that JSRV Env-induced cellular transformation was accompanied by a global transcriptomic shift.

To explore the biological implications of the common DEGs, we performed GO enrichment analysis focusing on the top 30 Biological Process terms. These results pointed to three major layers of cellular alteration. First, regarding cellular structure and microenvironment, terms such as cell periphery, plasma membrane, extracellular region, and extracellular space were prominently featured, indicating broad remodeling of membrane integrity and extracellular matrix secretion. Second, in the context of development, the coordinated presence of system development, anatomical structure development, and specifically epithelium development and epithelial cell differentiation, suggested that JSRV Env disrupts normal epithelial maturation programs. Third, terms related to signaling and stress—including signaling, response to stimulus, cell communication, and cell adhesion—highlighted a massive rewiring of intercellular networks and signal transduction (Fig. 2F).

KEGG pathway analysis further mapped these DEGs to several critical functional categories (Fig. 2G). Profound metabolic reprogramming was evident; the broad Metabolic pathways (notably glycolysis/gluconeogenesis and amino acid biosynthesis) showed the highest gene representation, pointing to a classic metabolic shift to fuel proliferation. This was accompanied by altered detoxification processes, as seen in the Metabolism of xenobiotics by cytochrome P450 and Bile secretion pathways. Carcinogenesis and stress response pathways were also highly represented. The co-occurrence of Chemical carcinogenesis - DNA adducts, Chemical carcinogenesis - reactive oxygen species, and Glutathione metabolism revealed oxidative DNA damage and a compromised antioxidant system, which was further supported by the activation of the HIF-1 signaling pathway for hypoxic adaptation. Crucially, the analysis highlighted the activation of core oncogenic cascades—including PI3K-Akt, MAPK, Hippo, Wnt, and JAK-STAT signaling—which collectively drive cell survival. Furthermore, viral-specific pathways (Viral protein interaction with cytokine and cytokine receptor and Viral carcinogenesis) overlapped with host Cytokine-cytokine receptor interaction, demonstrating how JSRV Env hijacks host communication networks. Finally, the presence of Autophagy - animal and Mitophagy - animal pathways suggested a mechanism for clearing stress-induced mitochondrial damage. Collectively, these interconnected pathways demonstrate that JSRV Env-induced transformation involves synergistic metabolic reprogramming, carcinogenic stress activation, and hijacking of signal transduction networks.

Furthermore, based on the results of KEGG signaling pathway analysis, the JSRV Env-induced transformation of BEAS-2B cells was found to trigger mitochondrial dysfunction and autophagy through the induction of energy metabolism disorders and oxidative stress.

**Fig. 3:**
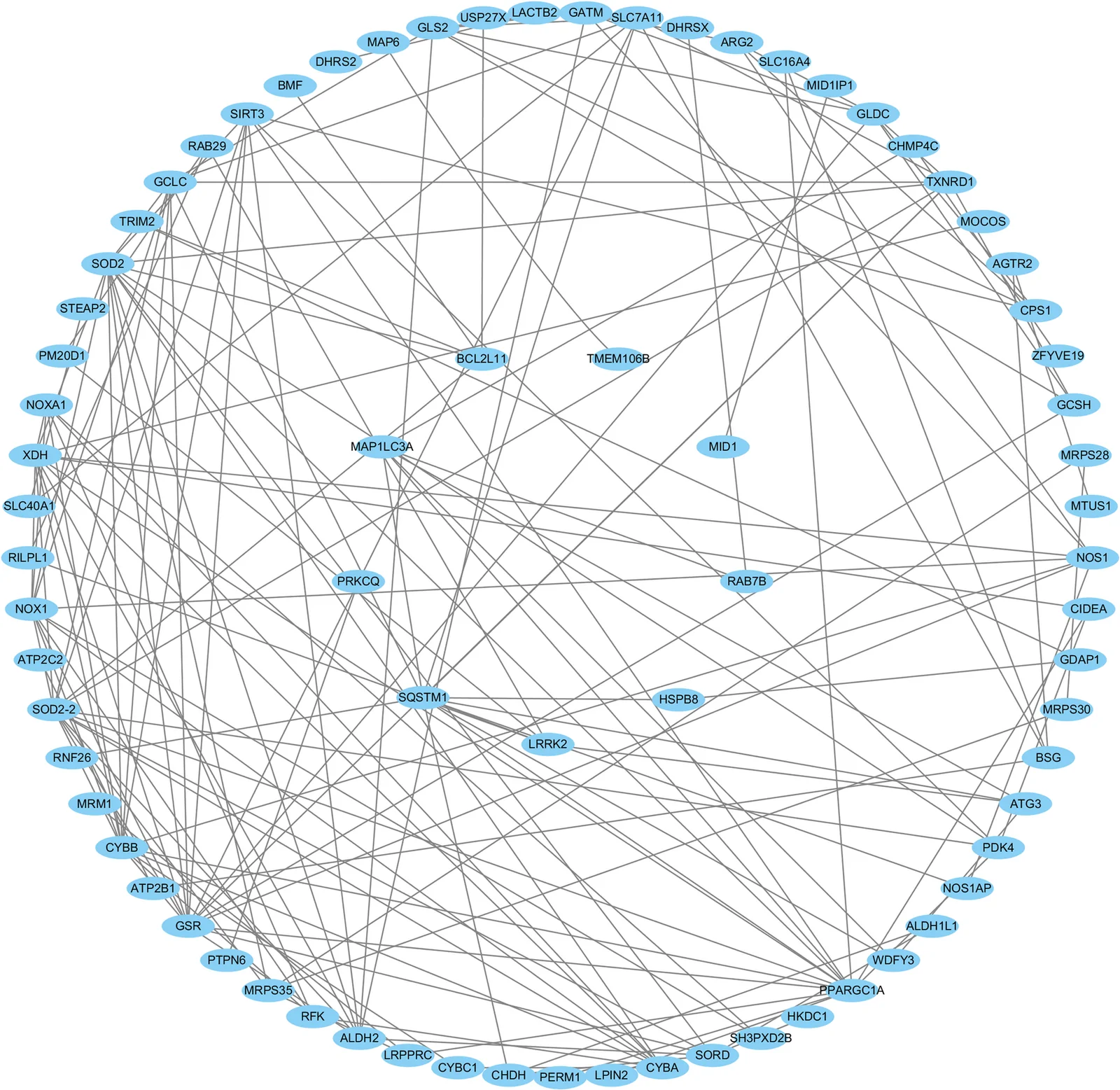
Potential regulatory network of mitochondria and genes related to mitophagy regulation. The outer circle represents mitochondria-related genes, and the inner circle represents genes related to mitophagy regulation

To visualize these intricate regulatory relationships, we constructed a correlation network encompassing key mitochondria-related genes and those related to mitophagy regulation. Using the Cytoscape software, we visualized regulatory network pairs with a Pearson correlation threshold greater than 0.8. The analysis revealed a network comprising 74 nodes connected by 156 edges, thereby elucidating the intricate associations between genes (Fig. 3).

### RT-qPCR -based confirmation of the transcriptome data

**Fig. 4:**
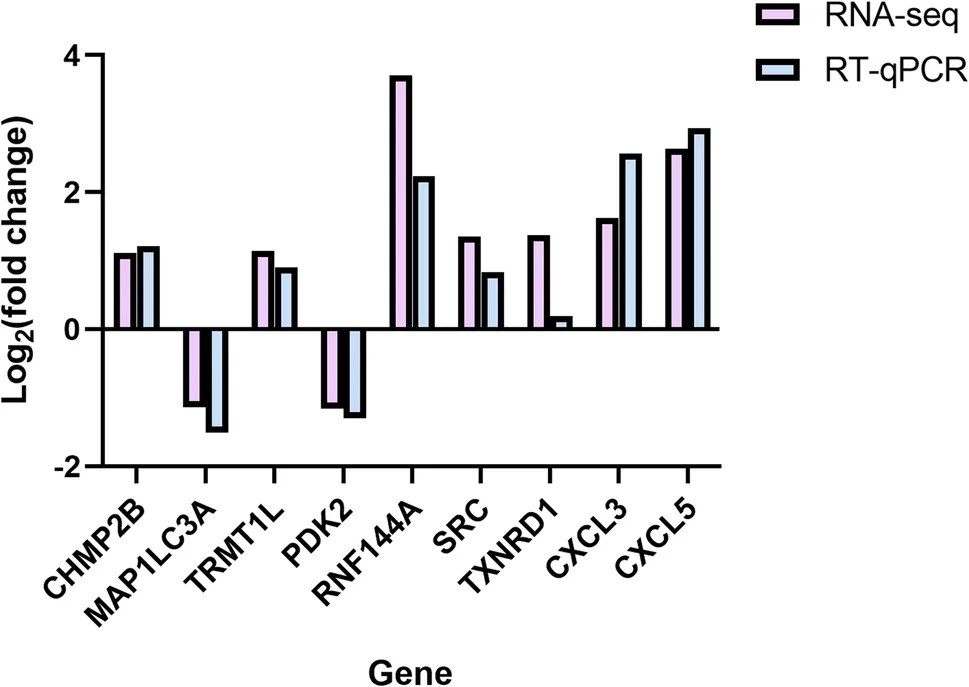
Validation of RNA-seq results by RT-qPCR

To validate the reliability of the RNA-seq data, RT-qPCR was performed to examine the expression of nine representative common DEGs, including CHMP2B, MAP1LC3A, TRMT1L, PDK2, RNF144A, SRC, TXNRD1, CXCL3, and CXCL5. These genes were selected from the common DEGs identified between the JSRV Env group and the two control groups. As shown in Fig. 4, the expression trends detected by RT-qPCR were consistent with those obtained from RNA-seq analysis, confirming the reliability of the transcriptomic data generated in this study.

### Metabolic profiling of JSRV Env-transformed BEAS-2B cells

**Fig. 5:**
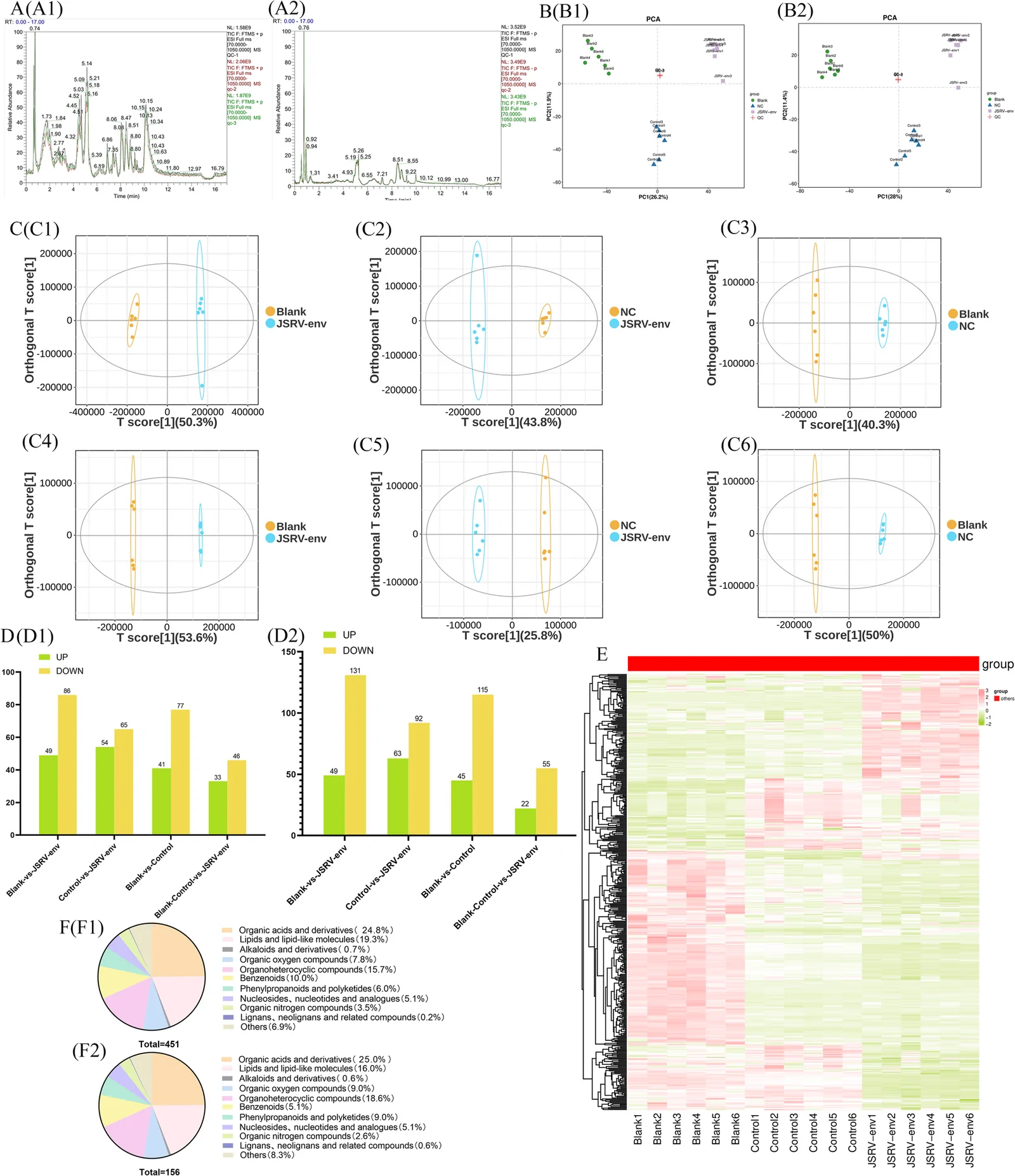
Identification and characterization of differentially expressed metabolites (DEMs) between the control and JSRV-env groups. **A** Total ion chromatogram of quality control (QC) sample mass spectrometry analysis (A1) Positive ion mode; (A2) Negative ion mode (**B**) Principal component analysis score plot (B1) Positive ion mode; (B2) Negative ion mode (**C**) Results of OPLS-DA analysis. C1–C3: Positive ion mode (C1: Blank vs. JSRV-env; C2: Negative control vs. JSRV-env; C3: Blank vs. Negative control); C4–C6: Negative ion mode (C4: Blank vs. JSRV-env; C5:Negative control vs. JSRV-env; C6: Blank vs. Negative control) (**D**) Differential metabolite analysis (D1) Bar chart analysis of DEMs in the positive ion mode; (D2) Bar chart analysis of DEMs in the negative ion mode; **E** Heat map of metabolite cluster analysis F Analysis results of different metabolite types (F1) Classification analysis results of all DEMs among the Blank, Control, and JSRV-env Groups (F2) Classification analysis results of common DEMs between the Blank/Control and JSRV-env groups. Abbreviations: JSRV, Jaagsiekte sheep retrovirus; Env, envelope

Cellular metabolites were profiled using HILIC-LC-MS in both the positive and negative ion modes. The high reproducibility of the QC sample total ion chromatograms confirmed the stability and reliability of the system (Fig. 5A1–A2). PCA showed distinct clustering of the Blank, negative control, and JSRV-*env* groups. PC1 and PC2 explained 38.1% of the variance in the positive mode and 39.4% in the negative mode (Fig. 5B1–2). Subsequently, OPLS-DA was used to enhance group separation. The resulting score plots revealed significant pairwise discrimination among the three groups in both ion modes (Fig. 5C1–6), confirming substantial metabolic differences and model robustness.

Based on the OPLS-DA model, metabolites with VIP > 1 and FC ≥ 2 or ≤ 0.5 were defined as DEMs. In total, 451 DEMs were identified across both ion modes (192 in positive ion mode and 259 in negative ion mode). A key finding of this study was the identification of a core metabolic signature that was consistently altered in the JSRV-env group relative to both the Blank and Negative controls. In the positive ion mode, this shared signature comprised 33 upregulated and 46 downregulated metabolites (Fig. 5D1). Similarly, in the negative ion mode, it consisted of 22 upregulated and 55 downregulated metabolites (Fig. 5D2).

Hierarchical clustering analysis of all DEMs revealed distinct metabolic signatures for each group as the samples were clustered according to their experimental classification (Blank, Control, and JSRV-*env*) (Fig. 5E). This clustering pattern visually confirms the significant metabolic alterations induced by JSRV-*env* expression. In total, 451 DEMs were identified across the Blank, Control, and JSRV-*env* group. Ranked by the number of metabolites in descending order, the categories were as follows: Organic acids and derivatives (24.8%), Organoheterocyclic compounds (15.7%), Lipids and lipid-like molecules (19.3%), Benzenoids (10.0%), Organic oxygen compounds (7.8%), Others (6.9%), Phenylpropanoids and polyketides (6.0%), Nucleosides, nucleotides, and analogues (5.1%), Organic nitrogen compounds (3.5%), Alkaloids and derivatives (0.7%), and Lignans, neolignans, and related compounds (0.2%) (Fig. 5F1).

Among the 451 DEMs, 156 were identified as common between the Blank/Control and JSRV-*env* groups. Ranked by the number of metabolites in descending order, the categories were as follows: Organic acids and derivatives (25.0%), Organoheterocyclic compounds (18.6%), Lipids and lipid-like molecules (16.0%), Organic oxygen compounds (9.0%), Phenylpropanoids and polyketides (9.0%), Benzenoids (5.1%), Nucleosides, nucleotides, and analogues (5.1%), Organic nitrogen compounds (2.6%), Alkaloids and derivatives (0.6%), and Lignans, neolignans and related compounds (0.6%) (Fig. 5F2).

### Analysis of DEMs

**Fig. 6:**
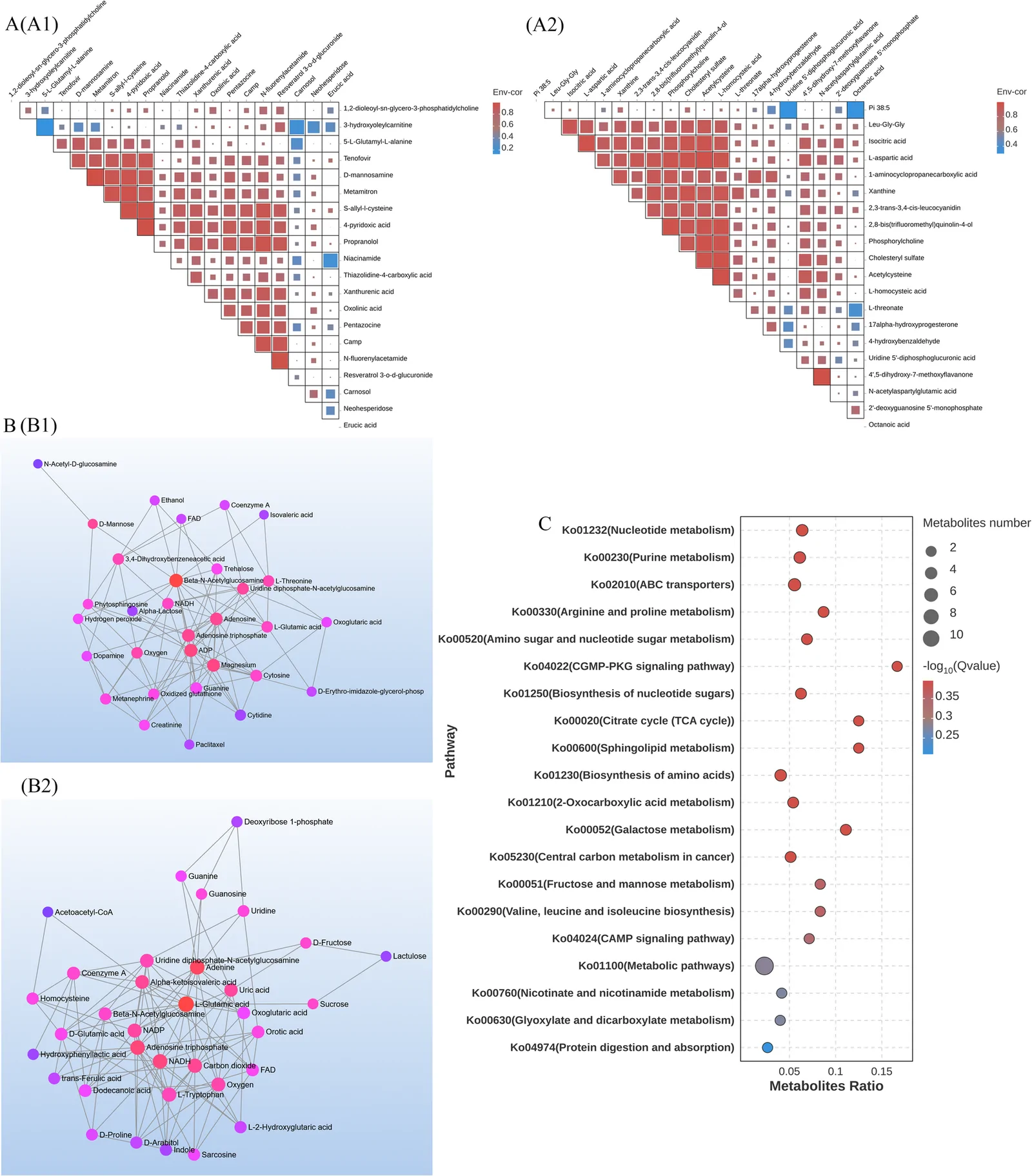
Analysis of DEMs between Blank and Control groups compared with those in the JSRV-*env* group.e (**A**) Correlation heat map analysis (A1) Positive ion mode; (A2) Negative ion mode (**B**) Interaction network diagram of differentially expressed metabolites (DEMs) (B1) Positive ion mode; (B2) Negative ion mode (**C**) Analysis of the metabolic pathways of DEMs. Abbreviations: JSRV, Jaagsiekte sheep retrovirus; Env, envelope

To investigate the interplay between metabolites altered by JSRV Env, the common DEMs in both the positive and negative ion modes were subjected to a correlation heatmap analysis. The analysis revealed distinct clusters of highly correlated metabolites, demonstrating that the JSRV Env transformation orchestrates broad, systematic metabolic reprogramming. In the positive ion mode (Fig. 6A1), these clusters encompassed a diverse range of biochemical classes, including lipids (e.g., 1,2-dioleoyl-sn-glycero-3-phosphocholine), amino acid derivatives (e.g., 5-L-Glutamyl-L-alanine), carbohydrates (e.g., D-mannosamine), and xenobiotics (e.g., tenofovir). A similar multipathway correlation pattern was observed in the negative ion mode (Fig. 6A2), with distinct clusters containing amino acids (e.g., L-aspartic acid), nucleotides (e.g., 2’-deoxyguanosine 5’-monophosphate), and lipids (e.g., phosphocholine). Collectively, these findings from both ion modes demonstrate the synergistic dysregulation of fundamental metabolic pathways, particularly amino acid and lipid metabolism. This coordinated disruption affects critical cellular processes including energy metabolism, biomacromolecular synthesis, signal transduction, and xenobiotic detoxification.

To further investigate the metabolic interactions underlying JSRV Env-mediated transformation, we constructed metabolite interaction networks from the common DEMs in both ion modes. These networks revealed a profound reprogramming of central metabolic hubs, providing a system-level view of cellular perturbations. A central finding was the coordinated dysregulation of energy and amino acid metabolism. Initially suggested by correlation analysis—where the TCA cycle intermediate isocitric acid strongly correlated with amino acid derivatives—this synergy was directly confirmed by the topological features of the networks. Specifically, in positive ion mode, the hub metabolite Beta-N-acetylglucosamine interacted with ATP and ADP (Fig. 6B1), while in negative ion mode, L-glutamic acid and adenine emerged as hubs connecting to ATP, NADH, and NADP (Fig. 6B2). This dual disruption of mitochondrial energy production and amino acid catabolism indicates a metabolic remodel to meet the bioenergetic and biosynthetic demands of malignant transformation. Beyond energy metabolism, the networks implicated other critical alterations. The consistent presence of nucleic acid metabolites (e.g., Cytidine, Guanine, Adenine) across both modes suggests interference with nucleotide synthesis and genomic homeostasis. Additionally, carbohydrate interactions (e.g., UDP-GlcNAc) pointed to reprogramming in glycolysis and hexosamine biosynthesis. Finally, the identification of stress-linked metabolites, such as phytosphingosine (sphingolipid signaling) and L-tryptophan (aryl hydrocarbon receptor signaling), indicated the activation of adaptive pathways to maintain homeostasis under metabolic stress. Collectively, these interaction networks unravel the multidimensional metabolic crosstalk driving JSRV Env-induced transformation.

Pathway enrichment analysis of the 156 common DEMs highlighted the systematic nature of this metabolic shift, identifying 56 significantly altered pathways (Fig. 6C). Core biosynthetic and energy-producing pathways—including Nucleotide metabolism, Purine metabolism, the Citrate cycle, and Sphingolipid metabolism—were the most significantly enriched, acting as primary nodes directly altered during transformation. Interestingly, the cGMP-PKG signaling pathway stood out: despite containing fewer DEMs, its exceptional statistical significance (low Q-value) positions it as a critical regulatory hub linking signal transduction to metabolic output. The broad scope of this reprogramming was visually reflected in the overarching “Metabolic pathways” category, which displayed the largest bubble size due to the integration of multiple metabolite classes. Furthermore, pathways such as ABC transporters, Arginine and proline metabolism, and Central carbon metabolism in cancer were significantly altered, suggesting contributory roles in sustaining the transformed phenotype. Collectively, these data delineate a multilayered metabolic network underlying JSRV Env-mediated transformation, spanning from core energy and biosynthetic pathways to specific regulatory signals.

### Validation of differential genes downstream of DEMs

**Fig. 7:**
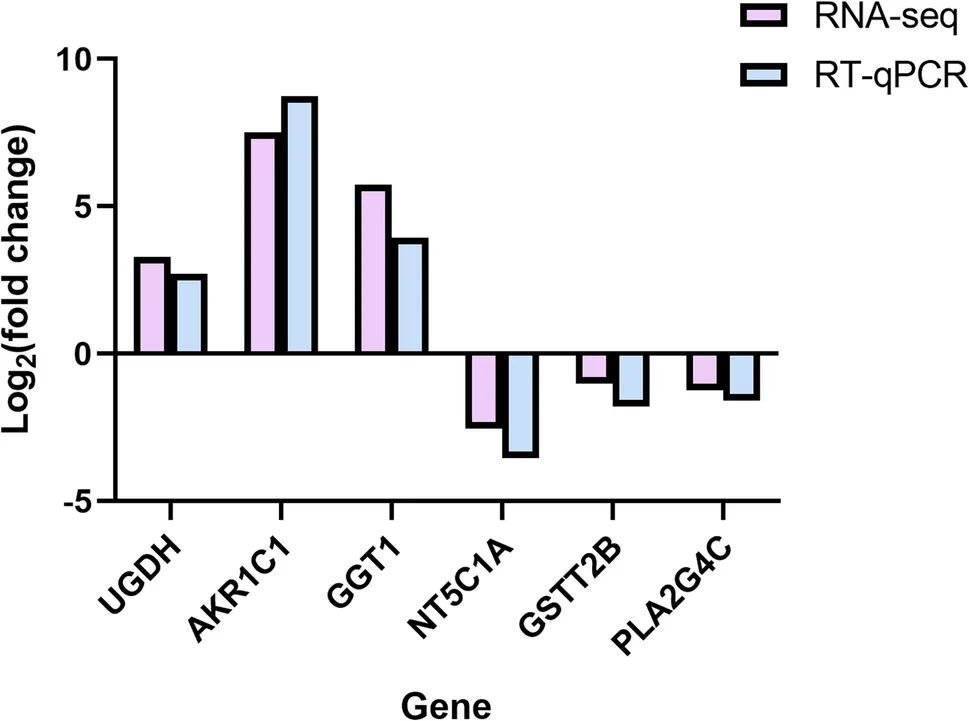
Concordance between RNA-seq and RT-qPCR results for key differentially expressed genes associated with differentially expressed metabolites

To further support the metabolomic findings and establish a multi-omics basis for subsequent mechanistic studies, RT-qPCR was performed to examine the expression of six key genes associated with representative gene–metabolite pairs. These pairs were selected because they were involved in several highly enriched and biologically relevant pathways. Specifically, GSTT2B and GGT1 were associated with oxidized glutathione and 5-L-glutamyl-L-alanine, respectively, both of which are related to redox homeostasis and glutathione metabolism. NT5C1A was linked to Ile-Pro in pyrimidine metabolism, while PLA2G4C was associated with 1,2-dihexadecanoyl-sn-glycero-3-phosphocholine in arachidonic acid metabolism. In addition, UGDH and AKR1C1 were related to amino sugar metabolism and steroid biosynthesis, respectively. As shown in Fig. 7, the expression trends of these six genes were generally consistent with the abundance changes of their corresponding metabolites, supporting the reliability of the metabolomic results and the biological relevance of the integrated multi-omics analysis.

### Results of pathway association analysis between RNA-Seq and metabolomics

**Fig. 8:**
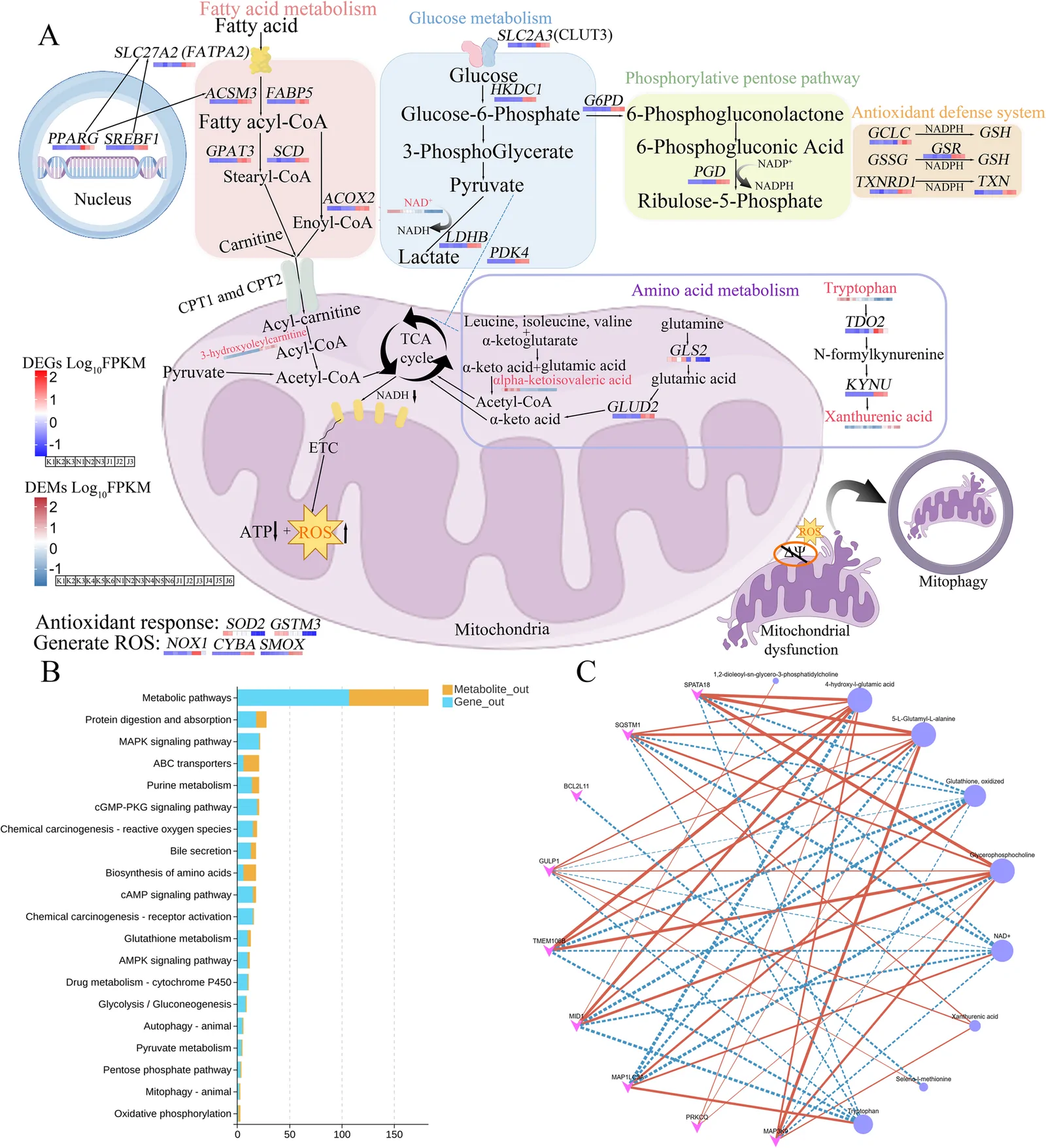
Correlation analysis between transcriptomics and metabolomics.** A** Multi-omics analysis reveals that JSRV Env drives the malignant transformation of BEAS-2B cells by inducing metabolic reprogramming characterized by enhanced glycolysis and lipid oxidation. This metabolic imbalance results in severe oxidative stress and mitochondrial damage, thereby activating compensatory mechanisms such as mitochondrial biogenesis and an integrated stress response (**B**) Joint analysis of Kyoto Encyclopedia of Genes and Genomes (KEGG) signaling pathways in transcriptomics and metabolomics (**C**) Interaction network of the key differential genes (DEGs) and differentially expressed metabolites (DEMs) related to mitophagy (Pink nodes: DEGs; Blue nodes: DEMs) Abbreviations: JSRV, Jaagsiekte sheep retrovirus; Env, envelope; BEAS-2B, human bronchial epithelial cells

Integrated pathway analysis of the transcriptomics and metabolomics data revealed synergistic regulation of genes and metabolites by JSRV Env, providing robust multi-omics evidence for the mechanism underlying the induction of malignant transformation in BEAS-2B cells (Fig. 8A). We identified DEGs and DEMs involved in critical pathways such as glycolysis (SLC2A3, HKDC1, PDK4), fatty acid metabolism (SLC27A2, 3-hydroxyoleylcarnitine), and amino acid metabolism (GLS2, GLUD2, TDO2, KYNU). Integrated analysis reveals that JSRV Env drives metabolic reprogramming by compromising mitochondrial OXPHOS while upregulating glycolysis and lipid β-oxidation. The resulting metabolic pressure induces oxidative stress and excessive ROS accumulation, causing mitochondrial injury. Consequently, when damage surpasses cellular repair limits, the mitophagy pathway is initiated to preserve intracellular homeostasis through the selective clearance of dysfunctional mitochondria (Fig. 8B).

Based on Pearson correlation analysis, we constructed a complex association network encompassing key mitophagy-related genes and metabolites. Focusing on 17 genes and 12 metabolites, this analysis revealed a network comprising 29 nodes interconnected by 167 edges, thereby elucidating the intricate associations between mitophagy-related genes and metabolites (Fig. 8C). These mitophagy-associated genes were strongly correlated with metabolites involved in energy substrate supply, mitochondrial energy coenzymes, metabolic reprogramming, and lipid homeostasis. For instance, NAD⁺ synthesis was robustly correlated with SQSTM1- and MAP1LC3A-mediated mitophagy, indicating that mitophagy plays a crucial role in maintaining basal energy metabolism. Collectively, these findings indicate that during BEAS-2B cell malignant transformation, JSRV Env disrupts the coordinated balance between energy metabolism and metabolic reprogramming by remodeling the associative relationships between mitophagy-related genes and metabolites. This not only enables cells to maintain a compensatory basal energy supply via mitophagy but also contributes to the progression from metabolic dysregulation to a malignant phenotype through bidirectional regulation between metabolites and genes.

Overall, these findings present a holistic pathway-level blueprint for the JSRV Env carcinogenic network by integrating metabolomic and transcriptomic data. Our approach identified co-enriched pathways and elucidated the mechanistic crosstalk between functional modules, such as energy reprogramming and oxidative stress. This integrated analysis, corroborated by molecular validation, provides a panoramic view of the cascading molecular events driving cellular transformation.

### Mitochondrial damage detection

**Fig. 9:**
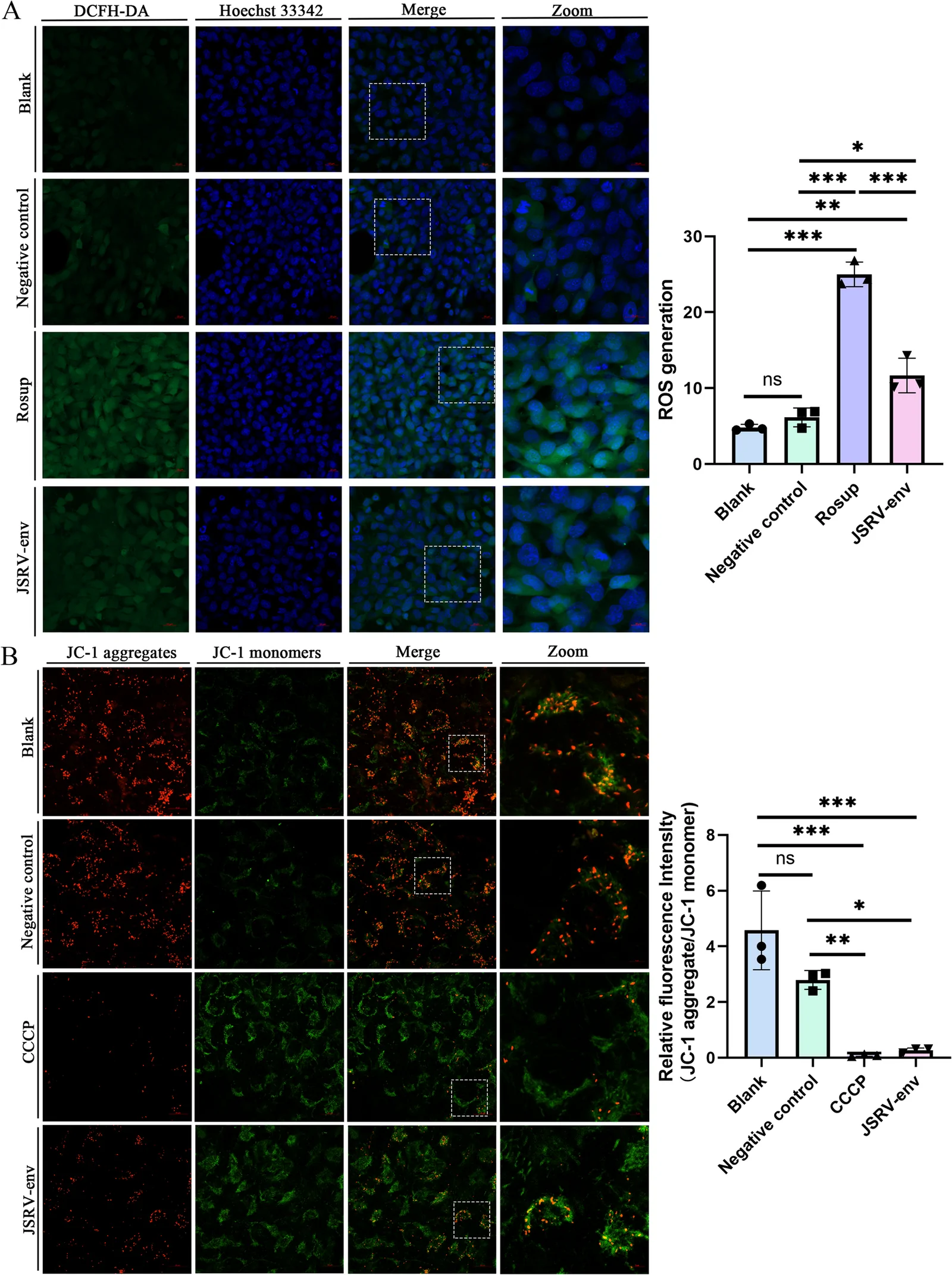
JSRV Env induces mitochondrial damage in transformed BEAS-2B cells.** A** Detection of reactive oxygen species (ROS) levels in JSRV Env-transformed BEAS-2B cells (Scale bar = 10 μm) (**B**) Detection of mitochondrial membrane potential (MMP) levels in JSRV Env-transformed BEAS-2B cells (Scale bar = 5 μm). *P* values were calculated using one-way analysis of variance. ^*^P < 0.05, ^**^P < 0.01, ^***^P < 0.001. Note: Blank group represents untreated BEAS-2B cells . Negative control group represents BEAS-2B cells transfected with empty vector. CCCP group or Rosup group represents BEAS-2B cells treated with CCCP (10 µM, 20 min) or Rosup (10 µM, 30 min). JSRV-*env* group represents BEAS-2B cells transfected with pcDNA4.0myc-his-JSRV-*env* plasmid. Abbreviations: JSRV, Jaagsiekte sheep retrovirus; Env, envelope; BEAS-2B, human bronchial epithelial cells

**Fig. 10:**
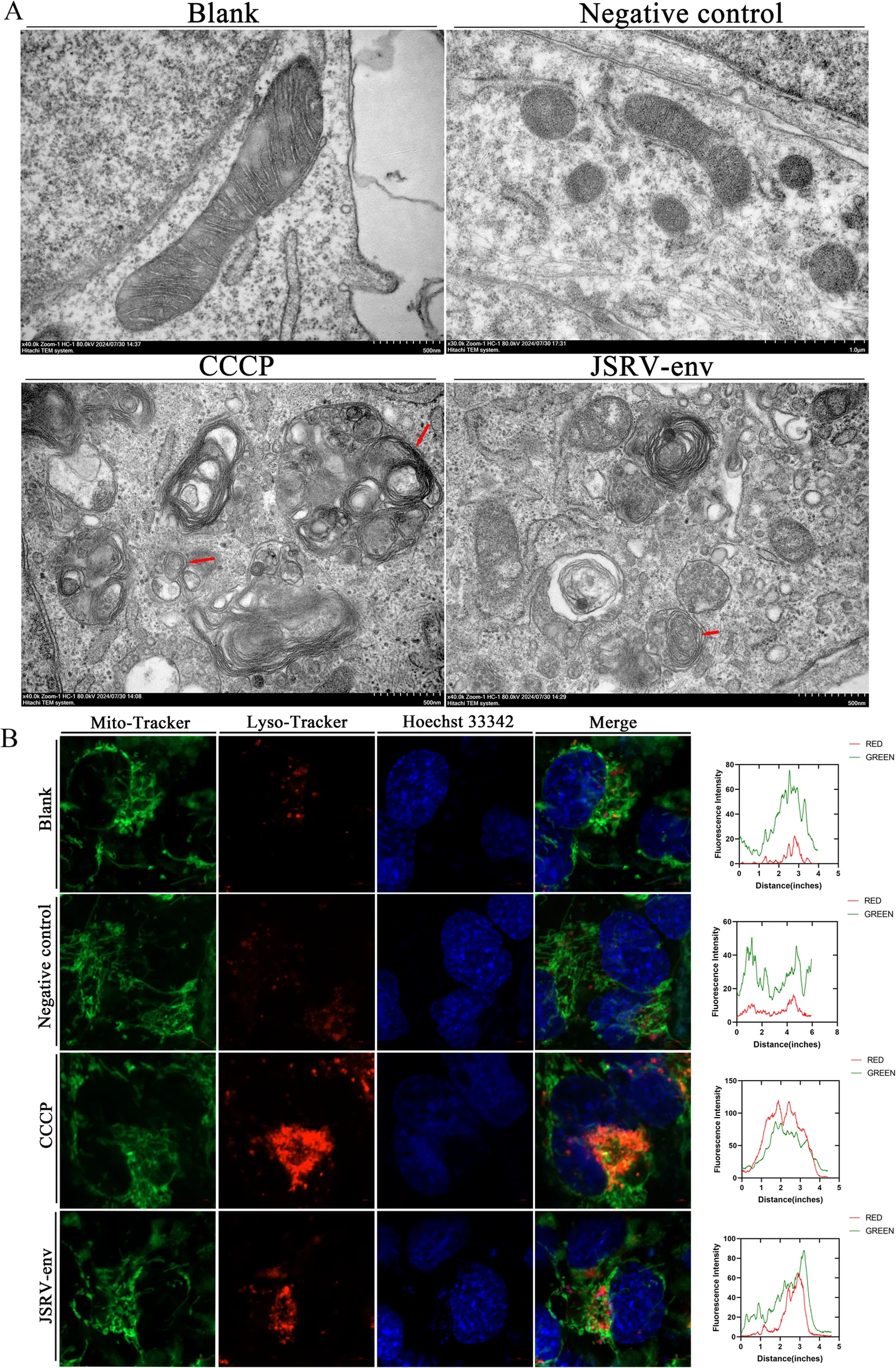
Detection of mitophagy in JSRV Env-transformed cells.** A** Observation results of mitochondria by TEM (Scale bar = 500 nm)(Red arrow indicates mitophagosome) (**B**) Mitochondrial and lysosomal probe co-localization assay (Scale bar = 5 μm). Note: Blank group represents untreated BEAS-2B cells. Negative control group represents BEAS-2B cells transfected with empty vector. CCCP group represents BEAS-2B cells treated with CCCP (10 µM, 20 min). JSRV-*env* group represents BEAS-2B cells transfected with pcDNA4.0myc-his-JSRV-*env* plasmid. Abbreviations: JSRV, Jaagsiekte sheep retrovirus; Env, envelope; BEAS-2B, human bronchial epithelial cells; TEM, transmission electron microscopy

To assess the mitochondrial damage induced by JSRV Env, we first measured intracellular ROS levels using DCFH-DA. Laser confocal microscopy revealed a significant increase in ROS fluorescence in JSRV Env-transformed BEAS-2B cells (Fig. 9A). Consistent with oxidative stress, JC-1 staining showed a reduced red/green fluorescence ratio, indicating enhanced MMP depolarization (Fig. 9B). Furthermore, TEM confirmed severe morphological damage, including mitochondrial swelling, cristae fragmentation, myelin-like lesions, and evidence of mitophagy (Fig. 10A). Finally, to quantify this clearance, we performed a co-localization analysis of mitochondria and lysosomes. The significantly increased overlap of their respective fluorescent signals confirmed that JSRV Env enhanced the lysosomal degradation of damaged mitochondria (Fig. 10B).

## Discussion

The reliability of our multi-omics approach is firstly supported by its successful recapitulation of established oncogenic mechanisms. Previous studies have demonstrated that JSRV Env induces malignant transformation in BEAS-2B cells primarily through the degradation of Hyal2, leading to the release and activation of RON and subsequent PI3K-Akt and MAPK signaling [20]. Notably, our transcriptomic data independently validated this core axis: RON (also known as macrophage-stimulating 1 receptor, which corresponded to MST1 in transcriptomic annotation) was significantly upregulated, and DEGs were prominently enriched in the PI3K-Akt and MAPK pathways. Furthermore, while Hyal2 exhibited a downward trend, it did not reach the strict screening threshold. Having confirmed the robustness of our model through these classical signaling events, we next leveraged the integrated transcriptomic and metabolomic data to systematically explore the broader, previously uncharacterized transcriptional and metabolic reprogramming induced by JSRV Env.

Viruses rely on host cell machinery to synthesize components for their own replication and assembly. Metabolic reprogramming induced by viral infection alters cellular metabolic patterns—such as glycolysis, amino acid metabolism, and lipid metabolism—to secure nutrients that support viral replication or rapid cell growth [21]. In our study, we found that JSRV Env drives the malignant transformation of BEAS-2B cells by inducing metabolic reprogramming, triggering oxidative stress, mitochondrial damage, and compensatory mitophagy. At the glycolytic level, JSRV Env upregulates genes such as SLC2A3, HKDC1, and PDK4, thereby enhancing glycolysis and inhibiting pyruvate entry into the mitochondria. In a study on hepatitis B virus infection, long non-coding RNA OIP5-AS1 led to the ubiquitination and degradation of the transcription factor sterol regulatory element binding protein 1, which regulates the transcription of HKDC1, thus inhibiting HKDC1 transcription and suppressing glycolysis to exert anti-tumor effects, demonstrating the necessity of the OIP5-AS1-HKDC1 axis in diagnosing and treating hepatitis B virus-induced liver cancer [22]. In West Nile virus (WNV) infection, glycolysis is upregulated and oxidative phosphorylation is downregulated. After treatment with the PDK4 inhibitor dichloroacetic acid, neuroinflammation caused by the WNV virus is alleviated [23]. This indicates that the regulators of the glycolytic pathway have therapeutic value in viral infectious diseases. Downregulation of NAD⁺ levels and accumulation of isocitrate in metabolites confirm this transformation, indicating impairment of OXPHOS function [24]. To compensate for this energy deficit, cells activated alternative pathways, among which a shift to lipid metabolism was particularly crucial. Lipid metabolic reprogramming reshapes energy supply, constructs signaling platforms, and provides membrane structures for rapidly proliferating cells [25]. In this study, the long-chain fatty acid intracellular transporter SLC27A2 transports long-chain fatty acids to mitochondria, enhancing the uptake of fatty acids; further, accumulation of the intermediate product 3-hydroxyacyl carnitine from β-oxidation confirms the enhancement of fatty acid β-oxidation, also reflecting the cellular dependence on lipid β-oxidation for energy supply after glucose metabolism disorder; in addition, upregulation of 1,2-dioleoyl-sn-glycero-3-phosphatidylcholine, the core phospholipid component of the mitochondrial membrane, is a compensatory synthesis of membrane lipids in cells in response to membrane damage and malignant transformation requirements [26]. This process may be regulated by multiple factors, such as the expression of SLC27A2 promoted by carcinoembryonic antigen-related cell adhesion molecule 6 in gastric cancer, thus promoting fatty acid uptake and promoting the tumor development process [27]. Although the detailed mechanism remains unclear in this study, other molecules involved in lipid metabolism, including ACSM3, FABP5, GPAT3, and SCD play crucial regulatory roles throughout the process, enhancing the malignant transformation ability of JSRV Env in terms of energy supply, biosynthesis, and optimization of membrane lipids.

In addition to glucose and lipid metabolism, amino acid metabolism has also undergone significant reprogramming. The reduced production of α-ketoisovalerate suggested inhibition of the branched-chain amino acid catabolic pathway [28]. Our data also revealed a profound rewiring of glutamine metabolism: downregulation of GLS2 indicated impaired conversion of glutamine to glutamate, while the upregulation of mitochondrial GLUD2 appeared to act as an alternative route to funnel glutamate into α-ketoglutarate to feed the TCA cycle [29]. Concurrently, tryptophan was largely diverted into the kynurenine pathway, as evidenced by tryptophan depletion alongside the upregulation of downstream metabolites. An increased kynurenine-to-tryptophan ratio is often associated with enhanced tumor invasiveness and immune evasion in viral infections [30]. This massive metabolic imbalance directly triggered severe oxidative stress. On one hand, the upregulation of NOX1, CYBA, and SMOX promoted excessive reactive oxygen species (ROS) generation, with SMOX being particularly associated with ROS accumulation and DNA damage in various cancers [31]. On the other hand, the downregulation of key antioxidant enzymes, such as mitochondrial SOD2 and GSTM3, coupled with decreased uric acid levels, collectively crippled the cellular antioxidant defense capacity [32, 33]. Notably, the upregulation of xanthine suggests simultaneous ROS production during purine synthesis [34]. Excessive ROS causes multi-dimensional damage to mitochondria, disrupting respiratory chain function and inducing mtDNA oxidation [35]. Moreover, excessive ROS production leads to the activation of the cellular antioxidant response. The antioxidant system is a complex network whose functions depend on the synergistic actions of various enzymes and metabolites. In addition to SOD2 and GSTM3, glutathione (GSH) and thioredoxin systems play core roles. Glutathione peroxidase uses reduced GSH to reduce hydrogen peroxide to water, whereas GSR regenerates GSH, a process highly dependent on NADPH. The TXNRD1 and thioredoxin systems also use NADPH to reduce oxidized proteins and eliminate ROS. GSR and TXNRD1 are considered to regulate tumor progression by modulating the antioxidant-reducing reaction in lung cancer [36].

Under these conditions, cells initiate a dual stress response. Initially, ells attempted mitochondrial repair and replenishment by upregulating PPARGC1A to activate mitochondrial biogenesis, serving as a positive compensatory response [37]. Subsequently, cells triggered a comprehensive stress response by upregulating stress-sensing genes such as SESN3, CHAC1, and TRIB3. SESN3, as a regulator, participates in cell protection and repair mechanisms by regulating cellular oxidative stress, covering antioxidant, anti-aging, and anti-tumor properties; further, its abnormal expression is closely related to cellular and oxidative stress, metabolic state, and specific signaling pathways [38]. CHAC1, as a γ-glutamyl cyclotransferase, affects calcium signal transduction and mitochondrial function by degrading glutathione, and is crucial for maintaining the redox balance in tumors and regulating cell death pathways, with potential as a biomarker and therapeutic target [39]. TRIB3 plays an important role in regulating the cell cycle, oxidative stress, signal transduction, inflammation, and immunity and is widely used in tumor research [40]. As indicated in this study, when cells respond to stress, SESN3, CHAC1, and TRIB3 may adapt to metabolic stress by regulating signaling pathways such as PI3K-Akt. However, when the degree of damage exceeds the repair capacity, cells are forced to activate the mitophagy pathway to selectively eliminate dysfunctional mitochondria and maintain basic homeostasis. Mechanistically, damaged mitochondria were targeted for autophagic clearance via PRKCQ-mediated phosphorylation of ULK1 [41]. SQSTM1/p62 binds to ubiquitinated outer mitochondrial membrane proteins via its UBA domain and simultaneously interacts with LC3 through its LIR domain to deliver damaged mitochondria to autophagosomes [42]. Upregulation of SQSTM1/p62 directly reflects an enhanced cellular capacity for ubiquitination-mediated mitophagy recognition. Additionally, the decreased protein level of MAP1LC3A reflects enhanced autophagosome-lysosome fusion and content degradation, indicating increased autophagic flux. Ultimately, PPARGC1A drives mitochondrial biogenesis to replenish new mitochondria, and achieve damage clearance and functional recovery [43]. This series of compensatory responses ultimately leads to significant upregulation of mitophagy, representing a core adaptive mechanism by which cells respond to energy metabolism disorders during malignant transformation. Although our integrated multi-omics approach and morphological observations—such as the transcriptomic alterations of key mitophagy-related markers (e.g., MAP1LC3A and SQSTM1) and the accumulation of autophagosomes and damaged mitochondria in TEM images—strongly suggest that mitophagy serves as a core mechanistic pathway in JSRV Env-induced transformation, we acknowledge that this remains an inference. A major limitation of the current study is the lack of direct biochemical and functional validation. Specifically, the definitive quantitative assessment of mitophagic flux (e.g., LC3-I/II conversion and p62 degradation) and functional intervention experiments (such as pharmacological inhibition or targeted gene knockdown) are still required. Future studies dedicated to these biochemical and functional validations will be essential to definitively establish the causal relationship and elucidate the precise mechanistic cascade.

Eventually, under the continuous action of JSRV Env, this imbalance between damage, compensatory repair, and compensatory clearance causes cells to chronically remain in a state of genomic instability and stress, forming the key molecular cascade driving malignant transformation. This finding is consistent with a study by Karakurt et al., which reported that ROS accumulation in OPA lung tissues leads to enhanced lipid peroxidation and DNA damage. Although the terminal lipid peroxidation product malondialdehyde (MDA) and the DNA damage marker 8-OHdG were not detected in our untargeted metabolomics analysis, this discrepancy is explainable. MDA readily reacts with proteins or DNA to form adducts, which may escape detection as unknown compounds in untargeted approaches, while 8-OHdG is often present at levels below the detection threshold of such platforms [44]. Concurrently, OPA-affected lung tissues have also exhibited upregulated cellular oxidation, inflammatory responses, and autophagy [45]. This study expands the understanding of JSRV pathogenesis by linking oxidative stress to cellular metabolic reprogramming and mitochondrial damage. These findings provide valuable insights and data, enriching the current knowledge base in JSRV research. However, the exact pathogenic mechanism of JSRV Env remains to be fully elucidated. Existing results suggest that JSRV Env does not mediate cell transformation through a single mechanism, but through a complex signal cascade reaction combined with regulatory networks of related signaling pathways, such as PI3K-Akt and MAPK, as well as metabolic-related molecules that directly or indirectly participate in regulating the signaling pathway network. Further, the metabolic state (such as energy level, redox state, and nutrient availability) directly affects signaling pathway activity, reshaping the inherent signal regulatory system within the cell in multiple ways. Currently, the absence of a suitable in vitro culture system for wild-type JSRV precludes the direct investigation of gene and metabolic variations via viral infection, necessitating the use of plasmid-based oncogene transfection. This constitutes a primary limitation, as other viral genomic structures may also play pivotal roles in cell transformation. Furthermore, the potential physiological impact of transfection reagents must be acknowledged; although screening for differential features common to both blank and negative control groups helps mitigate this confounding factor, it cannot be entirely eliminated, as the inherent cytotoxicity of these reagents also affects cellular physiology. Additionally, because this approach relies on transient transfection, it precludes in-depth investigation into the long-term effects of Env on cells. Subsequent research will focus on optimizing the JSRV transformation protocol to exclude extraneous variables, thereby enabling a more rigorous investigation into the long-term impact of Env on cellular homeostasis and providing a clearer explanation of the underlying pathogenic mechanisms.

## Conclusions

The findings of this study elucidate the pathogenic mechanisms of JSRV and provide new insights for the discovery of antiviral targets. The combined transcriptomic and metabolomic analysis of the key metabolic mechanisms of JSRV Env-transformed cells in this study provided an in-depth interpretation of core pathways such as glycolysis, amino acid metabolism, and lipid metabolism. This reveals the metabolic dependence of JSRV Env on cellular metabolic reprogramming, particularly in glycolysis and lipid metabolism. We also evaluated mitochondrial damage based on cellular oxidative stress and discovered that JSRV Env mediated mitophagy induction. Overall, this study offers a new metabolic perspective for understanding the pathogenic mechanism of JSRV and supplements the current research on JSRV Env-induced mitophagy, providing important insights for subsequent studies on the mechanism of JSRV-transformed cells. Building upon the present study, future research will delve deeper into the mechanisms of JSRV Env-mediated cell transformation, the critical pathways involved in metabolic reprogramming, and the precise mechanisms regulating mitophagy. Furthermore, we will continue to optimize the protocols for JSRV cell transformation to minimize interference from confounding factors.

## Supplementary Information

Supplementary Material 1.

Supplementary Material 2.

Supplementary Material 3.

Supplementary Material 4.

Supplementary Material 5.

## Data Availability

The RNA-seq data generated in this study have been deposited in the Sequence Read Archive (SRA) of the National Center for Biotechnology Information (NCBI) under the accession number SUB15906009 and the BioProject number PRJNA1397990.The metabolomics dataset generated in this study has been submitted to the China National Center for Bioinformation (CNCB) OMIX database under the accession number OMIX014227 and the BioProject number PRJCA055344.

## Abbreviations

JSRV: Jaagsiekte sheep retrovirus
Env: Envelope
BEAS-2B: Human bronchial epithelial cells
DEMs: Differentially expressed metabolites
DEGs: Differentially expressed genes
OPA: Ovine pulmonary adenocarcinoma
LUAD: Lung adenocarcinoma
RNA-seq: Transcriptome sequencing
RT-qPCR: Quantitative reverse transcription polymerase chain reaction
MDA: Malondialdehyde
ROS: Reactive oxygen species
WNV: West Nile virus
RON: Recepteur d’Origine Nantais
GO: Gene ontology
KEGG: Kyoto Encyclopedia of genes and genomes
GSH: Glutathione
FC: Fold change
QC: Quality control
MMP: Mtochondrial membrane potential
TEM: Transmission electron microscopy
PCA: Principal component analysis
Hpt: Hours post-transfection
OXPHOS: Oxidative phosphorylation
TCA: Tricarboxylic acid
TXNRD1: Thioredoxin reductase
GSR: Glutathione reductase
NOX1: NADPH oxidase 1
mtDNA: Mitochondrial DNA
NAD^+^: Nicotinamide adenine dinucleotide
CYBA: Cytochrome b-245α chain
SMOX: Spermine oxidase
SOD2: Superoxide dismutase 2
GSTM3: Glutathione S-transferase M3
8-OHdG: 8-hydroxy-2’-deoxyguanosine
