# Supplementary material for: Metabolic reprogramming, oxidative stress, and mitophagy in JSRV Env-transformed BEAS-2B cells: insights from integrated transcriptomics and metabolomics
Source: BMC Genomics. 2026 Jun 29;27:771. doi: 10.1186/s12864-026-13125-8 (PMC13591864; doi:10.1186/s12864-026-13125-8)
Supplement: Supplementary file 1 [file 12864_2026_13125_MOESM1_ESM.docx]

Table. S1 Sequences for *JSRV - env* detection

| Gene name | Accession number | Primer sequence(5′-3′) | Length/bp |
| --- | --- | --- | --- |
| *JSRV env* | JQ837489 | F:CTGCTTGGTCTAGGAATACTTGTG  R:TCCATTAGATGTTGGTGCTGTAAC | 155 |
| *β-actin* | NM_001101 | F:ATTGCCGACAGGATGCAGAA  R: CGGACTCGTCATACTCCTGC | 172 |
