# Supplementary material for: Metabolic reprogramming, oxidative stress, and mitophagy in JSRV Env-transformed BEAS-2B cells: insights from integrated transcriptomics and metabolomics
Source: BMC Genomics. 2026 Jun 29;27:771. doi: 10.1186/s12864-026-13125-8 (PMC13591864; doi:10.1186/s12864-026-13125-8)
Supplement: Supplementary file 2 [file 12864_2026_13125_MOESM2_ESM.docx]

Table.S2 Primer sequences for quantitative reverse transcription polymerase chain reaction (RT-qPCR)

| Gene name | Accession number | Primer sequence(5′-3′) | Length/bp |
| --- | --- | --- | --- |
| *CHMP2B* | NM_014043 | F:TGGCGTCCCTCTTCAAGAAG  R:AGCCCTCTGTGTACCTCGTA | 80 |
| *MAP1LC3A* | NM_032514 | F:GGTGATCATCGAGCGCTACA  R:ATGTTGACATGGTCCGGGAC | 84 |
| *TRMT1L* | NM_030934 | F:GAAACCTTGGCATAGTGTCAGTG  R:TCTGGCTGCTAGTTCCTTGTAAT | 131 |
| *PDK2* | NM_002611 | F:TCTCCATCCGCATGCTCATC  R:CACCTCAGAGACGTTGCAGT | 113 |
| *RNF144A* | NM_014746 | F:GCAATTAGCTGCCCAGATGC  R:TCCGACAGGGATCAAACAGC | 124 |
| *SRC* | NM_005417 | F:ACATCCCCAGCAACTACGTG  R:CAGTAGGCACCTTTCGTGGT | 163 |
| *TXNRD1* | NM_001093771 | F:AGGCTTATCAGGAGGGCAGA  R:GGACCATCACCTTCTTGCCA | 159 |
| *CXCL3* | NM_002090 | F:GTGAATGTAAGGTCCCCCGG  R:TTCTGAACCATGGGGGATGC | 107 |
| *CXCL5* | NM_002994 | F:TGTGTTGAGAGAGCTGCGTT  R:CGTTCTTCAGGGAGGCTACC | 134 |
| *β-actin* | NM_001101 | F:ATTGCCGACAGGATGCAGAA  R: CGGACTCGTCATACTCCTGC | 172 |
