# Supplementary material for: Metabolic reprogramming, oxidative stress, and mitophagy in JSRV Env-transformed BEAS-2B cells: insights from integrated transcriptomics and metabolomics
Source: BMC Genomics. 2026 Jun 29;27:771. doi: 10.1186/s12864-026-13125-8 (PMC13591864; doi:10.1186/s12864-026-13125-8)
Supplement: Supplementary file 3 [file 12864_2026_13125_MOESM3_ESM.docx]

Table.S3 Primer sequences for quantitative reverse transcription polymerase chain reaction (RT-qPCR)

| Gene name | Accession number | Primer sequence(5′-3′) | Length/bp |
| --- | --- | --- | --- |
| *UGDH* | NM_001184700 | F:AGAAGAGGTAGCAACAGCGATTG  R:GCTCCCACCAAACCCAACAC | 82 |
| *AKR1C1* | NM_001353 | F:ATGCCTGTCCTGGGATTTGG  R:AATCTTGCTTCGGATGGCCA | 162 |
| *GGT1* | NM_001288833 | F:GTACCACCGCATCGTAGAGG  R:GGTGAGTGGTGTCGTCAGAG | 158 |
| *NT5C1A* | NM_032526 | F:GAGAAGGCCCACGAGAACAA  R:GGTACGAATTGGGCACTCCA | 123 |
| *GSTT2B* | NM_001080843 | F:TGCCCGTGTTCATGAGTACC  R:TTGCGTTCCACCTTCTCCTC | 129 |
| *PLA2G4C* | NM_003706 | F:CCACAAGATCCCCTTTCCCC  R:ATGCATCACCACTGGTCCAG | 109 |
| *β-actin* | NM_001101 | F:ATTGCCGACAGGATGCAGAA  R:CGGACTCGTCATACTCCTGC | 172 |
