# Supplementary material for: Metabolic reprogramming, oxidative stress, and mitophagy in JSRV Env-transformed BEAS-2B cells: insights from integrated transcriptomics and metabolomics
Source: BMC Genomics. 2026 Jun 29;27:771. doi: 10.1186/s12864-026-13125-8 (PMC13591864; doi:10.1186/s12864-026-13125-8)
Supplement: Supplementary file 4 [file 12864_2026_13125_MOESM4_ESM.docx]

Preprocessing results of sequencing data quality

| Sample | Raw Reads（bp） | Clean Reads（bp） | Q20% | Q30% | GC% |
| --- | --- | --- | --- | --- | --- |
| Blank1 | 41155942 | 41103584 (99.87%) | 97.82% | 94.84% | 45.97% |
| Blank2 | 47058056 | 46927282 (99.72%) | 97.91% | 95.01% | 45.87% |
| Blank3 | 42610550 | 42562824 (99.89%) | 97.85% | 94.96% | 46.41% |
| Control1 | 39365922 | 39259290 (99.73%) | 98.18% | 95.60% | 45.06% |
| Control2 | 41586872 | 41496968 (99.78%) | 97.93% | 95.06% | 45.28% |
| Control3 | 37443126 | 37362404 (99.78%) | 98.09% | 95.45% | 45.88% |
| JSRV-*env*1 | 36732004 | 36691440 (99.89%) | 98.04% | 95.20% | 45.58% |
| JSRV-*env*2 | 42679640 | 42634862 (99.90%) | 98.16% | 95.50% | 45.74% |
| JSRV-*env*3 | 41822660 | 41772988 (99.88%) | 98.26% | 95.62% | 45.96% |

Compare the statistics of ribosomes

| Sample | Clean Reads（bp） | Mapped Reads(%) | Unmapped_Reads(%) |
| --- | --- | --- | --- |
| Blank1 | 41103584 | 336116 (0.82%) | 40767468 (99.18%) |
| Blank2 | 46927282 | 374420 (0.80%) | 46552862 (99.20%) |
| Blank3 | 42562824 | 380042 (0.89%) | 42182782 (99.11%) |
| Control1 | 39259290 | 250128 (0.64%) | 39009162 (99.36%) |
| Control2 | 41496968 | 250128 (0.67%) | 41217748 (99.33%) |
| Control3 | 37362404 | 249578 (0.67%) | 37112826 (99.33%) |
| JSRV-env1 | 36691440 | 282438 (0.77%) | 36409002 (99.23%) |
| JSRV-env2 | 42634862 | 359086 (0.84%) | 42275776 (99.16%) |
| JSRV-env3 | 41772988 | 355124 (0.85%) | 41417864 (99.15%) |

The results of valid read alignment to the genome sequence

| Sample | Clean Reads | Unmapped | Unique_Mapped | Total_Mapped |
| --- | --- | --- | --- | --- |
| Blank1 | 40767468 | 1334090 (3.27%) | 38448232 (94.31%) | 39433378 (96.73%) |
| Blank2 | 46552862 | 1479372 (3.18%) | 43962107 (94.43%) | 45073490 (96.82%) |
| Blank3 | 42182782 | 1298417 (3.08%) | 39833210 (94.43%) | 40884365 (96.92%) |
| Control1 | 39009162 | 1197437 (3.07%) | 36907503 (94.61%) | 37811725 (96.93%) |
| Control2 | 41217748 | 1368149 (3.32%) | 38885316 (94.34%) | 39849599 (96.68%) |
| Control3 | 37112826 | 1112262 (3.00%) | 35114650 (94.62%) | 36000564 (97.00%) |
| JSRV-env1 | 36409002 | 889821 (2.44%) | 34645801 (95.16%) | 35519181 (97.56%) |
| JSRV-env2 | 42275776 | 990548 (2.34%) | 40242957 (95.19%) | 41285228 (97.66%) |
| JSRV-env3 | 41417864 | 971433 (2.35%) | 39412228 (95.16%) | 40446431 (97.65%) |
